# Enumerateblood – an R package to estimate the cellular composition of whole blood from Affymetrix Gene ST gene expression profiles

**DOI:** 10.1186/s12864-016-3460-1

**Published:** 2017-01-06

**Authors:** Casey P. Shannon, Robert Balshaw, Virginia Chen, Zsuzsanna Hollander, Mustafa Toma, Bruce M. McManus, J. Mark FitzGerald, Don D. Sin, Raymond T. Ng, Scott J. Tebbutt

**Affiliations:** 1 PROOF Centre of Excellence, Vancouver, BC Canada; 2 BC Centre for Disease Control, Vancouver, BC Canada; 3 Division of Cardiology, University of British Columbia, Vancouver, BC Canada; 4 Department of Pathology and Laboratory Medicine, University of British Columbia, Vancouver, BC Canada; 5 Department of Computer Science, University of British Columbia, Vancouver, BC Canada; 6 Department of Medicine, Division of Respiratory Medicine, University of British Columbia, Vancouver, BC Canada; 7 Centre for Heart Lung Innovation, University of British Columbia, Vancouver, BC Canada; 8 Institute for Heart and Lung Health, Vancouver, BC Canada

## Abstract

**Background:**

Measuring genome-wide changes in transcript abundance in circulating peripheral whole blood is a useful way to study disease pathobiology and may help elucidate the molecular mechanisms of disease, or discovery of useful disease biomarkers. The sensitivity and interpretability of analyses carried out in this complex tissue, however, are significantly affected by its dynamic cellular heterogeneity. It is therefore desirable to quantify this heterogeneity, either to account for it or to better model interactions that may be present between the abundance of certain transcripts, specific cell types and the indication under study. Accurate enumeration of the many component cell types that make up peripheral whole blood can further complicate the sample collection process, however, and result in additional costs. Many approaches have been developed to infer the composition of a sample from high-dimensional transcriptomic and, more recently, epigenetic data. These approaches rely on the availability of isolated expression profiles for the cell types to be enumerated. These profiles are platform-specific, suitable datasets are rare, and generating them is expensive. No such dataset exists on the Affymetrix Gene ST platform.

**Results:**

We present ‘Enumerateblood’, a freely-available and open source R package that exposes a multi-response Gaussian model capable of accurately predicting the composition of peripheral whole blood samples from Affymetrix Gene ST expression profiles, outperforming other current methods when applied to Gene ST data.

**Conclusions:**

‘Enumerateblood’ significantly improves our ability to study disease pathobiology from whole blood gene expression assayed on the popular Affymetrix Gene ST platform by allowing a more complete study of the various components of this complex tissue without the need for additional data collection. Future use of the model may allow for novel insights to be generated from the ~400 Affymetrix Gene ST blood gene expression datasets currently available on the Gene Expression Omnibus (GEO) website.

**Electronic supplementary material:**

The online version of this article (doi:10.1186/s12864-016-3460-1) contains supplementary material, which is available to authorized users.

## Key Points

- We introduce a model that accurately predicts the cellular composition of blood from Affymetrix Gene ST gene expression profiles.
- This model outperforms existing methods when applied to Affymetrix Gene ST expression profiles from whole blood.
- The model is available on GitHub: https://github.com/cashoes/enumerateblood

## Background

Measuring genome-wide changes in transcript abundance in circulating peripheral whole blood cells is a useful way to study disease pathobiology [1]. By providing a relatively comprehensive survey of the status of the immune system, peripheral whole blood transcript abundances may help elucidate molecular mechanisms [2]. The sensitivity and interpretability of analyses carried out in this tissue, however, are significantly affected by its dynamic heterogeneity [3]. It is therefore desirable to quantify this heterogeneity, either to account for it or to model interactions that may be present between the abundance of certain transcripts, some cell types, and some phenotypic indication.

Accurate enumeration of the many component cell types that make up peripheral whole blood can be costly, however, and further complicates the sample collection process. Furthermore, the majority of publicly available peripheral whole blood-derived gene expression profiles on the Gene Expression Omnibus [4] do not include any composition information. Accurate quantification of the cellular composition of blood samples from gene expression data without performing additional experiments is useful, allowing for re-analysis of existing public data, for example.

Many approaches have been developed to infer the cellular composition of a sample from high-dimensional transcriptomic [3, 5–9] and, more recently, DNA methylation data [10, 11]. Briefly, if ***X***, ***W***, and ***H*** are matrices with entries ***X***_***ij***_ (observed expression for sample ***i***, gene ***j***), ***w***_***ik***_ (for sample ***i***, proportion of cell type ***k***), and ***h***_***kj***_ (cell type-specific contribution to the observed expression for cell type ***k***, gene ***j***), then the problem can be stated: having observed ***X***, we wish to estimate ***W***, based on the assumed relationship between expression and composition:

$$ {X}_{ij}={\displaystyle \sum_{k=1}^K{w}_{ik}{h}_{kj}+{e}_{ij}} $$

where *e*_*ij*_ represents the expression information for sample *i*, gene *j* that is not predictable by the cell composition.

We further assume that, for each component cell type ***k***, there exists a subset of features ***X*** ^***k***^_***ij***’_ in ***X*** whose observed expression in sample ***i*** is proportional to the relative abundance of cell type ***k*** in sample ***i***. More formally:

$$ {X}_{i{j}^{\prime}}^k\propto {w}_i^k $$

These composition-discriminating features are termed marker genes. For such genes, the elements of the ***H*** can be derived from e.g. omics profiles obtained from cells isolated from the tissue to be deconvolved (we refer to collections of such profiles as “reference datasets”), and ***W*** estimated by regression [5–11]. In this treatment, ***H*** is referred to as the basis matrix for deconvolution. We have previously used this approach to study cell-specific differential expression in the context of acute kidney allograft rejection, using GSE28490 as an Affymetrix U133 plus 2.0 “reference dataset” and identifying the basis matrix genes by using elastic net to derive a minimal multinomial classification model for the profiled cell types [12]. Importantly, mapping such marker genes across technology platforms is not always tractable. Not all genes can be readily mapped across gene expression platforms and the values derived from reference datasets may be specific to the platform on which the gene expression was measured. This limits application of these techniques to platforms on which suitable reference datasets exist. Unfortunately, generating such datasets is costly and replicating suitable existing studies on new platforms of limited scientific (and funding) interest. Reference datasets are correspondingly rare.

More recently, so-called reference-free approaches have been proposed to address this issue [13–15]. These approaches still require the identification of suitable marker genes for the cell types to be quantified, however, and this selection is of paramount importance to achieve optimal performance. The general strategy for marker selection is to identify genes whose expression in one cell type differs from that observed in all other cell types being considered [13], a process that itself relies on reference datasets. In fact, all approaches discussed thus far leverage one of a handful of publicly available reference datasets to derive a basis matrix or identify suitable marker genes [11, 16, 17]. No such suitable reference dataset exists on the newer Affymetrix Gene ST platform.

Here we apply a multi-task learning algorithm to construct a statistical model capable of predicting the composition of peripheral whole blood samples from Affymetrix Gene ST expression profiles. We demonstrate its performance on both Gene 1.0 ST (GEO platform GPL6244) and Gene 1.1 ST (GPL11532) datasets, which represent the bulk of the Gene ST arrays on GEO. Gene ST data summarized using custom CDF files (e.g. GPL16977, GPL15648 or GPL19370), or summarized to exon, rather than transcript, level (GPL10739), could be processed from the raw CEL files to a suitable format, though we did not evaluate performance in this case. We also show that the genes that make up this model can directly serve as marker genes, suggesting that it may be possible to identify marker gene sets for new technology platforms, or cell populations, using mixture gene expression profiles with corresponding cell composition information rather than using the more conventional reference dataset strategy.

The strategy we described in the current work could be readily applied to other tissues and/or platforms, which would allow for the development of tools to accurately segment and quantify a variety of admixed tissues from their gene expression profiles, to account for cellular heterogeneity across indications or model interactions between gene expression, some cell types and the indication under study. The described model is freely-available and open source, outperforms other current methods when applied to Gene ST data, and could significantly improve our ability to study disease pathobiology in blood by allowing a more complete study of the various components of the immune compartment of blood from whole blood gene expression.

## Methods

### Patient cohorts used

We used previously unpublished gene expression profiles from two large clinical cohorts to train and validate the new statistical model. The Rapid Transition Program (RTP) included prospectively enrolled patients with chronic obstructive pulmonary disease (COPD), presenting either to St. Paul’s Hospital or Vancouver General Hospital (Vancouver, Canada). Subjects presenting to the emergency department or those visiting the COPD clinic were approached for consent to participate in the study. Matched, peripheral blood derived, genome-wide transcript abundance and DNA methylation profiles were available for 172 blood samples from this cohort. The DNA methylation profiles were used to obtain estimates of the cellular composition of the blood samples, while the gene expression profiles were used to train a model to predict these inferred cell proportions and estimate its performance using cross-validation. Complete blood counts, including leukocyte differentials (CBC/Diffs) were available for all blood samples and used as an independent measure of blood composition (excluding lymphocyte subtypes).

The chronic heart failure (HF) program (CHFP) included prospectively enrolled HF patients presenting to St. Paul’s Hospital or Vancouver General Hospital (Vancouver, Canada). Subjects were approached during their visit to the heart function, pre-transplant, or maintenance clinics, and those who consented were enrolled in the study. A blood sample was collected at the time of enrollment. Genome-wide transcript abundance profiles and complete blood count, including leukocyte differential (CBC/Diffs) were available for 197 HF patients. This data was used to independently validate the performance of the statistical model.

The model’s performance was further validated using gene expression profiles obtained from a previously published asthma cohort (GSE40240) for which monocyte, B and T cell proportions were known [18, 19].

### Sample processing

The following describes sample processing for the RTP and CHFP cohorts. For details regarding sample processing for GSE40240 (asthma cohort) refer to Singh, et al. [18]. For all subjects, blood was collected in PAXgene (PreAnalytix, Switzerland) and EDTA tubes. The EDTA blood was spun down (200 x *g* for 10 min at room temperature) and the buffy coat aliquoted out. Both PAXgene blood and buffy coat samples were stored at −80 °C.

### Transcript abundance

Total RNA was extracted from PAXgene blood on the QIAcube (Qiagen, Germany), using the PAXgene Blood miRNA kit from PreAnalytix, according to manufacturer’s instructions. Human Gene 1.1 (GPL6244; RTP and CHFP cohorts) ST array plates (Affymetrix, United States) were used to measure mRNA abundance. This work was carried out at The Scripps Research Institute DNA Array Core Facility (TSRI; La Jolla, CA). The resulting CEL files were processed using the ‘oligo’ R package [20].

### DNA methylation

For the RTP cohort samples only, DNA was extracted from buffy coat using Qiagen’s QIAamp DNA Blood Mini kits. DNA was bisulfite-converted using the Zymo Research EZ DNA methylation conversion kit, and Infinium HumanMethylation450 BeadChips (Illumina, United States) were used to measure methylation status at >485,000 sites across the genome. This work was carried out at The Centre for Applied Genomics (TCAG; Toronto, Canada). The resulting IDAT files were processed using the ‘minfi’ R package [21].

### Statistical analysis

Following preprocessing with their respective packages (‘oligo’ or ‘minfi’), the normalized data were first batch corrected using the ‘ComBat’ algorithm [22], as implemented in the ‘sva’ R package [23]. A schematic representation of the statistical analysis is shown in Fig﻿. 1. 

#### Estimating cellular composition from DNA methylation profiles

Next, we inferred the cellular composition of the RTP cohort blood samples from their DNA methylation profiles using the ‘estimateCellCounts’ function provided by ‘minfi’. This function uses publicly available DNA methylation profiles obtained from isolated leukocyte sub-types to infer the relative abundance of granulocytes, monocytes, B, CD4+ T, CD8+ T and NK cells (details in Table 1) with very high accuracy [11, 21]. These estimates of the cellular composition of our training samples were used as a ‘silver standard’ to train the model in the absence of gold standard (e.g. flow cytometry) data to provide us with a ground truth. In order to gain additional confidence in these estimates, we compared them to those obtained from a hematology analyzer (CBC/Diffs) to assess accuracy and tested the 600 CpG sites used by ‘estimateCellCounts’ for associations with age, sex, or disease status in our training cohort (after adjusting for cellular composition using the CBC/Diffs) to determine whether these factors could be introducing any bias into the predictions.

**Table 1:** Description of predicted leukocytes

Cell name	Abbreviation used	Description
Granulocytes	Gran	CD15+ granulocytes
Monocytes	Mono	CD14+ monocytes
B cells	Bcell	CD19+ B-lymphocytes
T cells (CD4+)	CD4T	CD3 + CD4+ T-lymphocytes
T cells (CD8+)	CD8T	CD3 + CD8+ T-lymphocytes
NK cells	NK	CD56+ Natural Killer (NK) cells

**Fig. 1:**
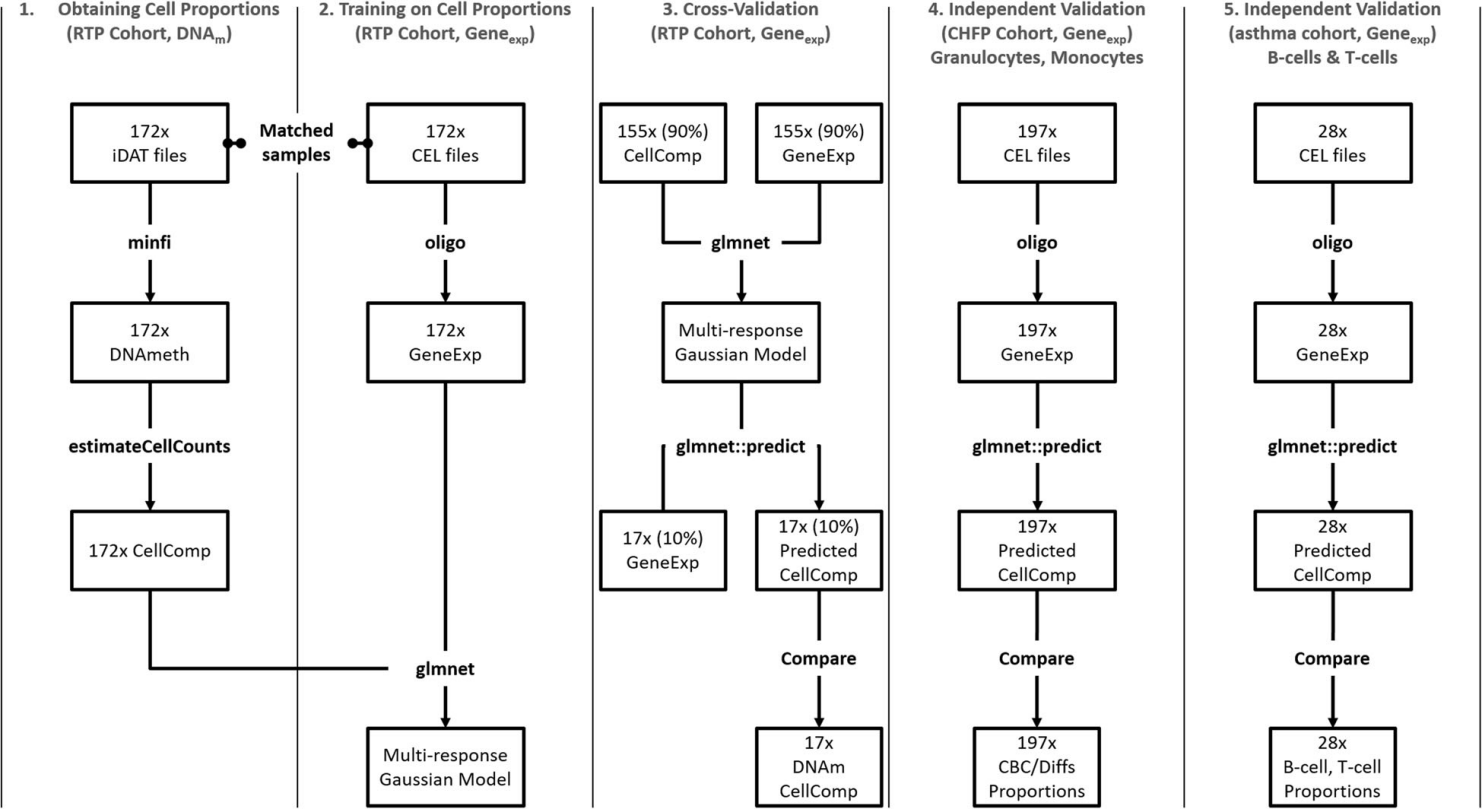
Schematic representation of the experiment. Cell proportions were estimated from DNA methylation profiles for 172 samples (1; COPD patients). These DNA methylation-derived cell proportions were used as a ‘silver’ standard in the absence of the ground truth. This ‘silver standard’ dataset was used to train a multi-response Gaussian model using the gene expression data (2). Out-of-sample performance was evaluated using a repeated (20x) 10-fold cross-validation (3) and in two independent sets of samples, in different clinical indications: granulocyte, monocyte, and lymphocyte performance was evaluated in a large set of samples (4; 197 samples, heart failure), while monocyte, B, and T cell prediction performance was evaluated in a smaller set (4; 28 samples, asthma)

#### Model training

We then fit a multi-response Gaussian model using elastic net regression via the ‘glmnet’ R package [24] on the genome-wide transcript abundance data, using the DNA methylation-derived cell proportions as response variables. The multi-response Gaussian model family is useful when there are a number of possibly correlated responses — a so called “multi-task learning” problem — as is the case for these cell proportions. Sparsity was an additional requirement: using a minimal set of features to predict cell proportions is desirable because it reduces the risk of bias being introduced under varied experimental conditions. Conversely, redundancy in the information provided by the features ensures robustness to such bias when it is present. We chose to use ‘glmnet’ because it is amenable to multi-task learning problems (using family = ‘mgaussian’) and can provide a balance between sparse models and redundancy in the model features (by tuning the parameter α), but any machine learning method that meets these requirements could potentially have been substituted.

Probesets with minimum log_2_ expression < 5.5 across all samples (22,251) were first excluded using the ‘exclude’ parameter. Next, we performed hyper-parameter tuning by running the ‘cv.glmnet’ function for a number of different values of α (α = 1, 0.9, 0.5, 0.1, 0), letting ‘cv.glmnet’ construct models using a sequence of λ values (default behavior). Briefly, the elastic net mixing parameter α provides a way to tune between ridge (α = 0) and lasso (α = 1) penalized regression, and the complexity parameter λ a means of adjusting the degree of shrinkage being applied to the coefficients. The optimal value for each parameter was that which minimized out-of-sample error rate in cross-validation, using the mean-square error criterion (‘cv.glmnet’ parameter type.measure = ‘mse’).

#### Estimating out-of-sample performance

Out-of-sample performance of our model was first evaluated using 10-fold cross-validation, repeated 20 times to eliminate any potential biases introduced by the partitioning of the data. We then validated the accuracy and calibration of our model by comparing its predicted proportions to that obtained from CBC/Diffs data in the CHFP cohort. Unfortunately, a more complete enumeration of the lymphocyte compartment (e.g., by flow cytometry) was not available in this large cohort, so we could not independently validate performance in the various lymphocyte sub-types. Instead, the sum of the predicted B, CD4+ T, CD8+ T and NK cell proportions was compared to total lymphocyte proportions from the CBC/Diffs. In addition, we accessed a small asthma cohort from GEO (GSE40240) for which monocyte, B- and pan T-cell (CD3+) proportions were available (Epiontis qPCR cell quantification assay) [19]. This dataset was used to independently validate the performance of the model’s B- and T-cell predictions.

In all cases, we report both model error (root-mean-square error) and correlation (Pearson’s product–moment correlation) to the actual cell proportions. The latter is more pertinent, however, as accurate multivariate calibration is not necessary for our intended use for these predicted proportions, namely as proxy measures useful for statistical work in the absence of more direct, clinically relevant, measures.

#### Performance compared to the method described in Abbas et al.

We also compared the performance of our model to an alternative approach for determining the composition of blood samples from their gene expression profiles, described in Abbas et al. [5], in both the CHFP and asthma cohorts. The basis matrix from Abbas et al., derived from the IRIS (Immune Response In Silico) reference dataset [16], was used to predict the cell proportions of neutrophils, monocytes, B, CD4+ T, CD8+ T and NK cells. These predicted proportions were compared to those obtained from CBC/Diffs (CHFP), or an Epiontis qPCR cell quantification assay (asthma), as above.

#### Model features as marker genes for the reference-free approach described in Chikina et al.

Finally, we evaluated whether our approach could be used to identify more suitable marker gene sets compared to using a reference dataset obtained on a different platform. The reference-free approach described by Chikina et al. [13], does not require a basis matrix, relying instead on a set of putative marker genes. These are used to guide the decomposition of the dataset’s covariance structure into separate variance components, using singular value decomposition. Marker genes for each cell type are summarized in this manner, a technique known as eigengene summarization [25]. Given a good set of marker genes, these summarized values, termed surrogate proportion variables (SPVs), should track with mixture proportions. Because SPVs are estimated directly in the dataset, platform mapping issues should be minimized, but whether marker genes identified on one platform may perform well when applied to another has not been evaluated before.

We used the reference-free approach described by Chikina et al. (as implemented in the ‘CellCODE’ R package) and marker genes derived either from the IRIS reference dataset, as recommended by Chikina et al., or the model features. Features were deemed marker genes for specific cell types based on the absolute value of their coefficient weights across cell types in the model. We then compared the SPVs produced by ‘CellCODE’, using either marker gene sets, to the CBC/Diffs, as above. Pearson’s product–moment correlation (r) was used to summarize association between predictions.

## Results

DNA methylation-derived predictions of the cellular composition of the RTP cohort blood samples were accurate when compared to those obtained from CBC/Diffs (root mean squared error [RMSE] = 0.01 – 0.08, Pearson’s r = 0.87 – 0.96; Additional file 1: Figure S1). The observed error rates for granulocytes and monocytes were consistent with those previously reported [10, 11]. We could not determine the accuracy of the B, CD4+ T, CD8+ T and NK cell predictions directly. The sum of these predicted proportions were well correlated to the lymphocyte proportions obtained from the CBC/Diffs (Pearson’ r = 0.87), however. When controlling for cellular composition using the CBC/Diffs, none of the 600 CpG sites used by ‘estimateCellCounts’ were significantly associated with disease status, but 8 were significantly associated with sex, and 70 with age, in our data (Additional file 2: Tables S1-S3).

**Fig. 2:**
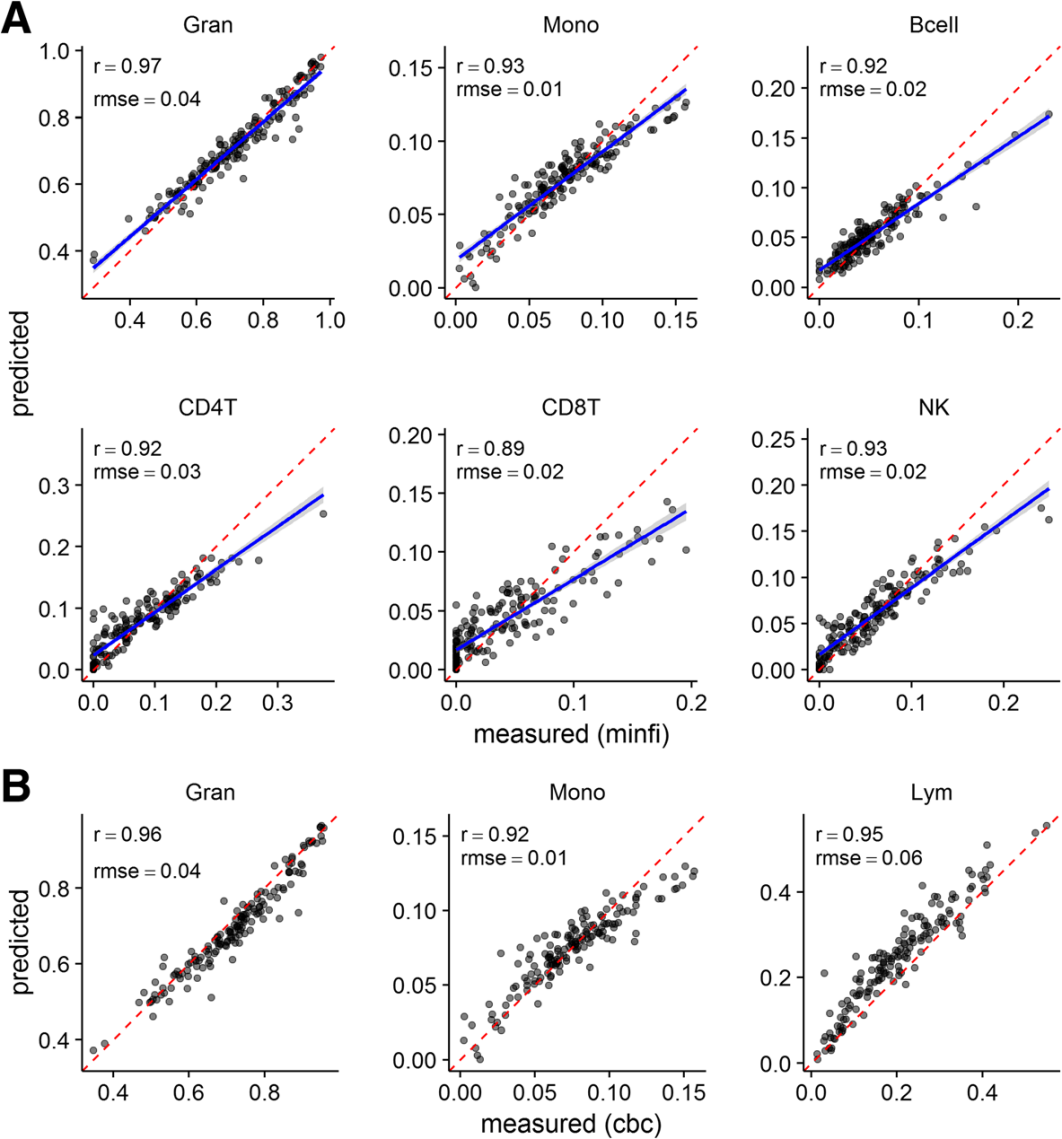
Assessing model fit. Predicted proportions from the model are plotted against the DNA methylation-derived cell proportions for each sample in the training data (**a**) or that obtained from CBC/Diffs (**b**). For (**a**), linear best-fit line to the data is plotted (blue line) with 95% point-wise confidence interval for fit (grey band) and compared with perfect agreement (red dashed line). For (**b**), predicted monocyte proportions are compared directly to the CBC/diffs. The predicted granulocyte proportions are compared to the sum of neutrophil, eosinophil and basophil proportions from the CBC/diffs, while the sum of the predicted B, CD4+ T, CD8+ T and NK cell proportions is compared to the total lymphocyte proportions from the CBC/diffs. For each cell type, Pearson’s product–moment correlation (Pearson’s r) and the root mean squared error (RMSE) are reported

These predictions were used as the response variables to train a multi-response Gaussian model in the RTP cohort gene expression data using elastic net regression. The optimal model hyper-parameterization (α = 0.1, λ = 0.8857) retained 491 features. Its fit to the data is visualized in Fig. 2, against both the DNA methylation derived composition estimates (Fig. 2a), and CBC/Diffs (Fig. 2b). Model fit was good across all cell types, with the exception, perhaps, of CD8+ T cells. When considering the model fit to the CBC/Diffs data, we noted slight bias, with granulocyte proportions tending to be under-predicted and lymphocyte proportions over-predicted.

**Fig. 3:**
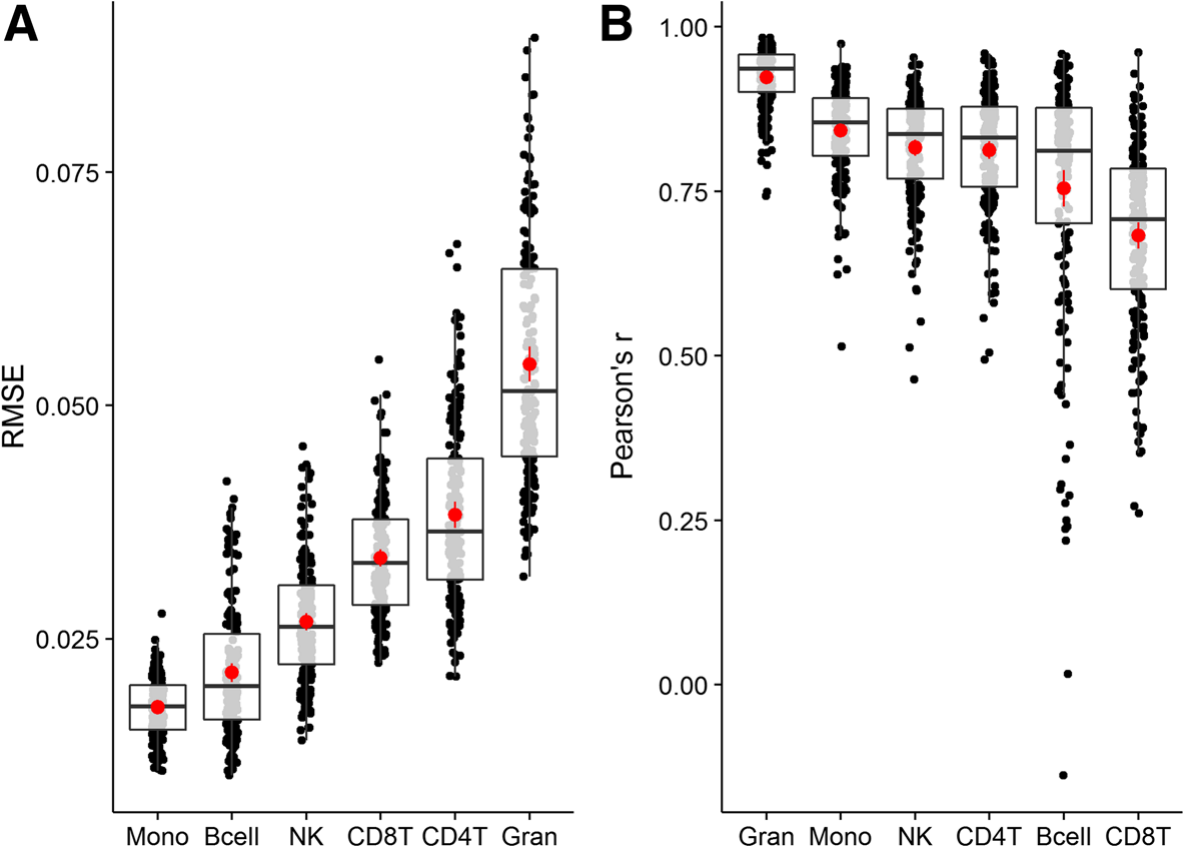
Cross-validation performance. Distribution of root mean square error (RMSE; (**a**)) and Pearson’s product–moment correlation (Pearson’s r; (**b**)) for out-of-sample predictions across repeated (20x) 10-fold cross-validations are visualized using boxplots. The mean and 95% CI are shown as a point and range in the center of each boxplot and represent the expected out-of-sample performance

To characterize the potential performance of this model on new data, we carried out a 20 × 10-fold cross-validation. We summarize the RMSE and Pearson’s r observed across 200 (20 × 10) left-out sets in Table 2 and the data is visualized in Fig. 3. Estimated out-of-sample performance varied across cell types; RMSE was lowest for monocytes and highest for granulocytes. Error rates compared favorably to other methods for inferring cellular composition of samples from gene expression data [5–7, 12]. Correlation between predicted and actual in the 200 left-out sets was highest for granulocytes (0.926), followed by monocytes (0.824), NK cells (0.812), CD4+ T cells (0.785), B cells (0.731), and CD8+ T cells (0.671).

**Table 2:** Model performance

Cell type	20x 10-fold cross-validation		Independent test set	
	RMSE (mean ± sd)	Pearson’s r (mean ± sd)	RMSE (n)	Pearson’s r (n)
Bcell	0.021 ± 0.007	0.755 ± 0.188	0.04 (28)	0.93 (28)
CD4T	0.038 ± 0.01	0.813 ± 0.09	0.06 (28)	0.91 (28)
CD8T	0.034 ± 0.006	0.683 ± 0.138		
Gran	0.054 ± 0.013	0.923 ± 0.046	0.06 (197)	0.89 (197)
Mono	0.018 ± 0.003	0.842 ± 0.068	0.02 (197)	0.74 (197)
NK	0.027 ± 0.006	0.816 ± 0.083	NA	NA

**Fig. 4:**
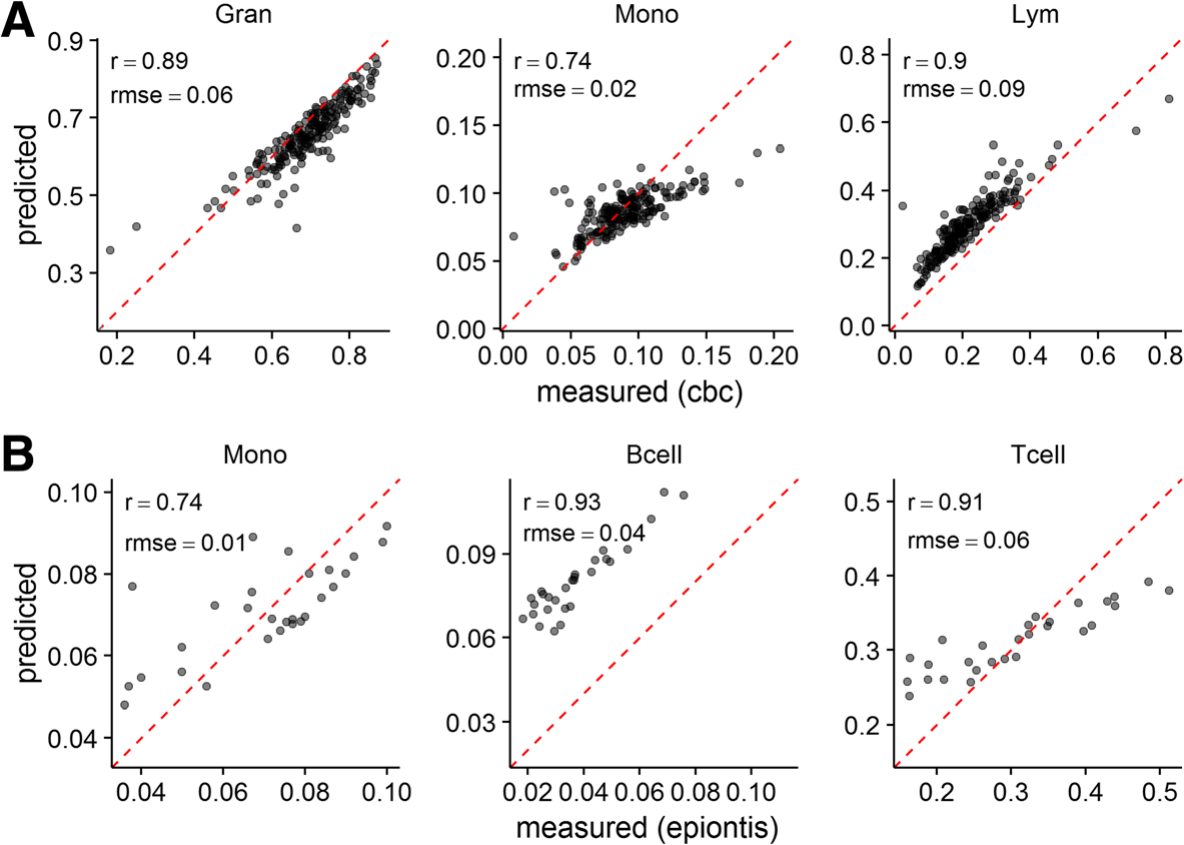
Our model accurately predicts the cellular composition of blood samples and outperforms existing approaches in Affymetrix Gene ST data. Predicted cell proportions are plotted against the cell proportions obtained from CBC/diffs ((**a**); CHFP cohort) or a cell-type specific DNA methylation cell-typing assay ((**b**); Epiontis asthma cohort). In (**a**), the sum of the predicted B, CD4+ T, CD8+ T and NK cell proportions is compared to the total lymphocyte proportions from the CBC/diffs. The predicted granulocyte and monocyte proportions are directly compared. In (**b**), the sum of the predicted CD4+ and CD8+ T cell proportions is compared to T cell proportion from the Epiontis assay. The predicted monocyte and B cell proportions are directly compared. For each cell type, Pearson’s product–moment correlation (Pearson’s r) and the root mean squared error (RMSE) are reported

Next, we applied the model to gene expression profiles from the CHFP (Fig. 4a) and asthma cohorts (Fig. 4b) in order to independently validate its performance. Performance in these cohorts is summarized in Table 2. The predicted proportions for granulocytes and monocytes were well correlated with those obtained from CBC/Diffs across all 197 samples in the CHFP cohort (r = 0.89 and 0.74 respectively; Fig. 4a). While we could not determine the accuracy of the B, CD4+ T, CD8+ T and NK cell predicted proportions directly, the sum of these predicted proportions were well correlated to the lymphocyte proportions obtained from the CBC/Diffs (r = 0.90). Monocyte, B and pan T-cell proportions were known in the smaller asthma cohort (Epiontis qPCR cell quantification assay [19]). There, B-cell predicted proportions were highly correlated to those obtained from the Epiontis assay (Fig. 4b). Again, we could not determine the accuracy of the CD4+ T and CD8+ T cell predicted proportions directly, but the sum of these predicted proportions was well correlated to the pan T cell proportions obtained from the Epiontis assay (r = 0.91). We also applied the basis matrix described by Abbas and others in [5] to predict cell proportions in both the CHFP and asthma cohorts (Additional file 3: Figure S2) by mapping the Affymetrix U133 identifiers to the corresponding Gene 1.1 (CHFP) or 1.0 (asthma) ST identifiers using the Biomart service [26, 27]. Our model generally performed better, especially for monocytes, though this was expected given that the Abbas, et al. basis matrix was developed on the Affymetrix U133 platform.

**Fig. 5:**
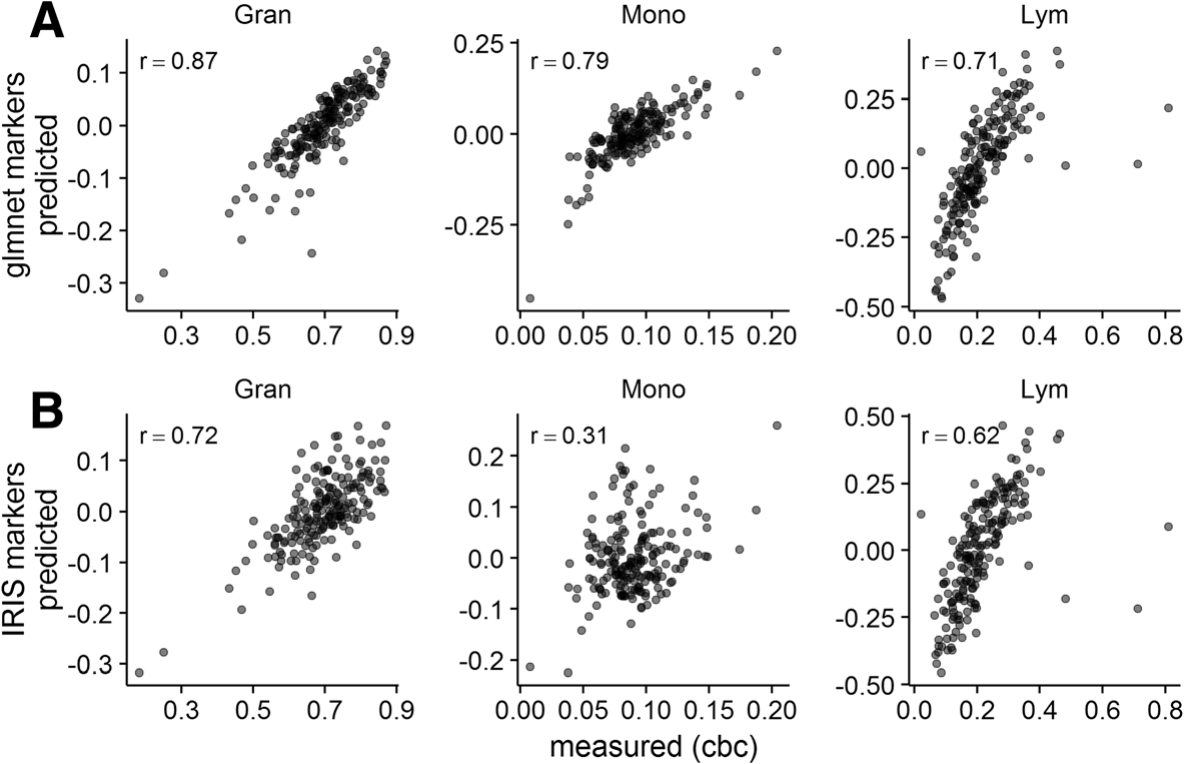
Our model identifies better performing marker genes for use with reference-free approaches in Affymetrix Gene ST data. Surrogate proportion variables obtained from CellCODE are plotted against the cell proportions obtained from CBC/Diffs in an independent dataset (CHFP cohort). The sum of the surrogate proportion variables obtained for B, CD4+ T, CD8+ T and NK cells is compared to the total lymphocyte proportions from the CBC/Diffs. Marker genes used by CellCODE were derived from the coefficients of the model (**a**) or using the recommended set of marker genes (**b**) derived from the IRIS reference dataset. For each cell type, Spearman’s rank correlation (ρ) is reported

More recently, reference-free approaches to quantifying the composition of mixed tissue samples from gene expression [13] or DNA methylation [14] profiles have been proposed. Such approaches may offer a solution to the platform mapping issues we describe. Reference-free approaches rely on the availability of marker genes for the cell types to be quantified. Many strategies have been described for identifying such marker genes [5, 12, 13, 28], but, so far, all have leveraged existing reference datasets: collections of gene expression profiles derived from cells isolated from the mixed tissue to be quantified. In order to determine whether marker gene selection exhibits platform-bias, we compared CellCODE SPVs derived using either marker genes identified from the IRIS reference dataset (U133 platform; mapped to Gene ST platform identifiers) or the features in our model. Marker genes derived from our model outperformed those identified from the IRIS reference dataset and mapped to Gene ST platform identifiers, when used with the CellCODE approach (Fig. 5). Interestingly, the marker gene sets showed minimal overlap (granulocytes = 3/51, monocytes = 4/58, B cells = 0/55, CD4+ T cells = 0/11, CD8+ T cells = 1/15, NK cells = 6/22). We confirmed that the genes in our model varied significantly across the included cell types by performing ANOVA in the GSE28490 dataset, which includes replicate profiles of the relevant cell types isolated from blood. Most (461/491; 94%) mapped identifiers varied across cell types (adjusted *p*-value < 0.05).

**Fig. 6:**
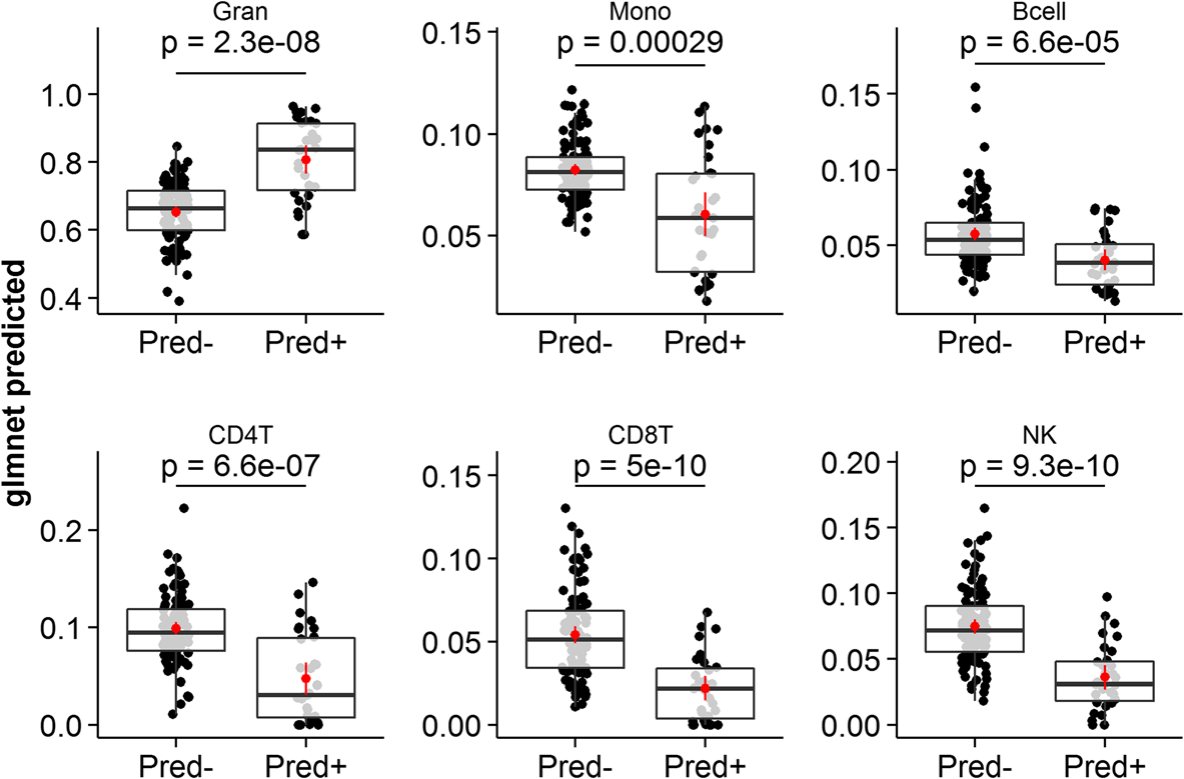
Model predicted cell proportions highlight prednisone-dependent changes in peripheral blood composition. Treatment of acute exacerbations (AE) in COPD with prednisone results in important changes in the cellular composition of peripheral blood. The distributions of granulocyte, monocyte, B, CD4+ T, CD8+ T and NK cell proportions are visualized for patients from the Rapid Transition Program (RTP) cohort that were given prednisone or not (*p*-value is for the unpaired Student’s *t*-test comparing the two groups in each case)

Finally, we applied the model to predict the composition of the RTP cohort blood samples from their gene expression. This is a contrived example, as this information was already available to us, but it serves to illustrate a possible application of the approach: to adjust for the confounding effect of changes in cellular composition when studying the effect of prednisone on whole blood gene expression. As expected, we observed large differences in the predicted proportions of the various cell types between patients given prednisone or not (Fig. 6). Patients on prednisone had proportionally lowered monocytes, B, CD4+ T, CD8+ T, and NK cells, and proportionally elevated granulocytes. This was consistent with the CBC/Diffs, and 460/491 genes in our model showed no significant residual association between their expression and prednisone status (adj. p-value > 0.05), after adjusting for cellular composition using the CBC/Diffs, suggesting that the observed differences reflect true changes in the cellular composition of the samples in response to prednisone, rather than changes in the gene expression of the underlying model features.

## Discussion

We introduce ‘Enumerateblood’, a freely available and open source R package that exposes a statistical model for predicting the composition of blood samples from Affymetrix Gene ST gene expression profiles. We demonstrate that this model has suitable performance across all included cell types in cross-validation, and validate its performance in two independent cohorts. The training and validation cohorts represent two major clinical indications, COPD and HF, and include patients with various comorbidities, on various medications, some with strong effects on blood gene expression (e.g., prednisone), suggesting that our model may generalize well and be broadly applicable. All training and validation samples were from older individuals, however, and it may be that this model will not generalize well to pediatric populations. A loss of performance in pediatric populations has been noted when using a similar approach with DNA methylation data [29].

We also show that platform-specific marker gene sets can be derived without the need for “reference datasets”: collections of gene expression profiles obtained from the isolated cells types we wish to enumerate. Using the model features as marker genes in combination with the reference-free approach proposed by Chikina et al. resulted in better performance compared to using marker genes derived from isolated leukocyte gene expression profiles obtained on another microarray platform. Interestingly, the reference-free approach performed only slightly worse than our model, although with loss of scale. This suggests that the coefficient weights of the model may be estimated directly in the data, and that these marker genes may be context-independent surrogates of cell proportions.

More generally, the strategy we adopted to train our model and identify suitable marker genes could be readily applied to other platforms, or tissues of interest. The only requirements are accurate quantification of the cell types of interest across a large cohort with matched omics profiling. For many popular platforms (e.g., RNA-seq), this schema may be more cost effective than sorting and profiling a number of replicates for all cells of interest, particularly when we consider how costs would scale with additional cell types to be quantified. Moreover, for low abundance cell types (e.g. Tregs), obtaining a sufficient quantity to profile may not be feasible, depending on the efficiency of available separation techniques, and amount of admixed tissue that can be collected in practice.﻿ Single cell RNA-seq may change all this in the near future, however.

The lack of independent validation for some of the lymphocyte sub-types (CD4+ T, CD8+ T, and NK cells) is a limitation, though cross-validation performance was good across all cell types. We believe it is unlikely that poor performance in some or all lymphocyte sub-types would result in good performance when summed and compared to CBC/Diffs. Model fit exhibits some degree of shrinkage (flattening of the plot of predicted vs. observed away from the 45 degree line). This is expected, however, and related to the phenomenon of regression to the mean. Performance in cross-validation was notably worse for CD8+ T cells. This could be because of the preponderance of zero values for this particular cell type. We also note that performance in monocytes drops significantly in the validation cohort. It is unclear why this is, but one possibility is the difference in the distribution of values in the validation cohort (mean monocyte proportion in training: 0.073 vs. 0.090 in the validation; *p =* 1.39 × 10^−7^). We have observed poor performance of various deconvolution approaches in quantifying monocytes in the past [12, 30]. It might be that circulating monocyte diversity is poorly reflected in our current framework and we may be selecting poor marker genes for this cell type as a result. A similar rationale could be applied to explain the poor CD8+ T cell performance results in cross-validation. Certainly, it offers the opportunity for further exploration of the true complexity of these cell types in peripheral blood.

## Conclusion

In summary, our freely-available and open source R package, ‘Enumerateblood’, exposes a statistical model capable of accurately inferring the composition of peripheral whole blood samples from Affymetrix Gene ST expression profiles. The strategy we adopted to derive this model is readily applicable to other tissues and/or platforms, which would allow for the development of tools to accurately segment and quantify a variety of admixed tissues from their gene expression profiles, to account for cellular heterogeneity across indications or model interactions between gene expression, some cell types and the indication under study. The described model outperforms other current methods when applied to Gene ST data. By allowing a more complete study of the various components of the immune compartment of blood from whole blood gene expression, this model will significantly improve our ability to study disease pathobiology in blood, and may generate novel insights from existing Affymetrix Gene ST blood gene expression datasets.

## Additional files

Additional file 1: Figure S1. DNA methylation-derived composition vs. CBC/Diffs. Predicted proportions were obtained by applying the ‘estimateCellCounts’ function from the ‘minif R package to peripheral blood derived DNA methylation profiles in the Rapid Transition Program (RTP) cohort and plotted against cell proportions obtained from CBC/Diffs. The sum of the predicted B, CD4+ T, CD8+ T and NK cell proportions is compared to the total lymphocyte proportions from the CBC/Diffs. For each cell type, Spearman’s rank correlation (ρ) and the root mean squared error (RMSE) are reported. (PNG 47 kb)
Additional file 2: Association of the 600 CpG sites used by minfi's ‘estimateCellCounts’ function with disease status (**Table S1**), sex (**Table S2**), or age (**Table S3**), after adjusting for cellular composition using CBC/Diffs in the RTP cohort. (XLSX 172 kb)
Additional file 3: Figure S2. Performance of Abbas et al. method in our validation datasets (Gene ST). Predicted cell proportions (using the method from Abbas et al.) are plotted against the cell proportions obtained from CBC/diffs (A; CHFP cohort) or a cell-type specific DNA methylation cell-typing assay (B; Epiontis asthma cohort). In A, the sum of the predicted B, CD4+ T, CD8+ T and NK cell proportions is compared to the total lymphocyte proportions from the CBC/diffs. The predicted granulocyte and monocyte proportions are directly compared. In B, the sum of the predicted CD4+ and CD8+ T cell proportions is compared to T cell proportion from the Epiontis assay. The predicted monocyte and B cell proportions are directly compared. For each cell type, Pearson’s product–moment correlation (Pearson’s r) and the root mean squared error (RMSE) are reported. (PNG 83 kb)

## References

[1] Chaussabel D (2015). Assessment of immune status using blood transcriptomics and potential implications for global health. Semin Immunol.

[2] Li S, Rouphael N, Duraisingham S, Romero-Steiner S, Presnell S, Davis C (2013). Molecular signatures of antibody responses derived from a systems biology study of five human vaccines. Nat Immunol.

[3] Shen-Orr SS, Gaujoux R (2013). Computational deconvolution: extracting cell type-specific information from heterogeneous samples. Curr Opin Immunol.

[4] Barrett T, Wilhite SE, Ledoux P, Evangelista C, Kim IF, Tomashevsky M (2013). NCBI GEO: archive for functional genomics data sets—update. Nucleic Acids Res.

[5] Abbas AR, Wolslegel K, Seshasayee D, Modrusan Z, Clark HF. Deconvolution of Blood Microarray Data Identifies Cellular Activation Patterns in Systemic Lupus Erythematosus. Tan P, editor. PLoS ONE. 2009;4:e6098. 10.1371/journal.pone.0006098 PMC2699551 19568420

[6] Gaujoux R, Seoighe C. Semi-supervised Nonnegative Matrix Factorization for gene expression deconvolution: a case study. Infect Genetics Evol. 2011;12:913–21. 10.1016/j.meegid.2011.08.014 21930246

[7] Gong T, Hartmann N, Kohane IS, Brinkmann V, Staedtler F, Letzkus M (2011). Optimal deconvolution of transcriptional profiling data using quadratic programming with application to complex clinical blood samples. PLoS One.

[8] Lu P, Nakorchevskiy A, Marcotte EM (2003). Expression deconvolution: a reinterpretation of DNA microarray data reveals dynamic changes in cell populations. Proc Natl Acad Sci U S A.

[9] Newman A, Liu C, Green M, et al. Robust enumeration of cell subsets from tissue expression profiles. Nat Methods. 2015;12:453–457. 10.1038/nmeth.3337 PMC4739640 25822800

[10] Houseman EA, Accomando WP, Koestler DC, Christensen BC, Marsit CJ, Nelson HH (2012). DNA methylation arrays as surrogate measures of cell mixture distribution. BMC Bioinformatics.

[11] Jaffe AE, Irizarry RA (2014). Accounting for cellular heterogeneity is critical in epigenome-wide association studies. Genome Biol.

[12] Shannon CP, Balshaw R, Ng RT, Wilson-McManus JE, Keown P, McMaster R (2014). Two-Stage, In Silico Deconvolution of the Lymphocyte Compartment of the Peripheral Whole Blood Transcriptome in the Context of Acute Kidney Allograft Rejection. PLoS ONE.

[13] Chikina M, Zaslavsky E, Sealfon SC (2015). CellCODE: a robust latent variable approach to differential expression analysis for heterogeneous cell populations. Bioinformatics.

[14] Houseman EA, Molitor J, Marsit CJ (2014). Reference-free cell mixture adjustments in analysis of DNA methylation data. Bioinformatics.

[15] Houseman EA, Kile ML, Christiani DC, Ince TA, Kelsey KT, Marsit CJ (2016). Reference-free deconvolution of DNA methylation data and mediation by cell composition effects. BMC Bioinformatics.

[16] Abbas AR, Baldwin D, Ma Y, Ouyang W, Gurney A, Martin F (2005). Immune response in silico (IRIS): immune-specific genes identified from a compendium of microarray expression data. Genes Immun.

[17] Allantaz F, Cheng DT, Bergauer T, Ravindran P, Rossier MF, Ebeling M (2012). Expression Profiling of human immune cell subsets identifies miRNA-mRNA regulatory relationships correlated with cell type specific expression. PLoS One.

[18] Singh A, Yamamoto M, Kam SHY, Ruan J, Gauvreau GM, O’Byrne PM, et al. Gene-Metabolite Expression in Blood Can Discriminate Allergen-Induced Isolated Early from Dual Asthmatic Responses. Hsu Y-H, editor. PLoS ONE. 2013;8:e67907. 10.1371/journal.pone.0067907 PMC3699462 23844124

[19] Singh A, Yamamoto M, Ruan J, Choi JY, Gauvreau GM, Olek S (2014). Th17/Treg ratio derived using DNA methylation analysis is associated with the late phase asthmatic response. Allergy, Asthma Clin Immunol.

[20] Carvalho BS, Irizarry RA (2010). A framework for oligonucleotide microarray preprocessing. Bioinformatics.

[21] Aryee MJ, Jaffe AE, Corrada-Bravo H, Ladd-Acosta C, Feinberg AP, Hansen KD (2014). Minfi: a flexible and comprehensive bioconductor package for the analysis of infinium DNA methylation microarrays. Bioinformatics.

[22] Johnson WE, Li C, Rabinovic A (2007). Adjusting batch effects in microarray expression data using empirical Bayes methods. Biostatistics.

[23] Leek JT, Johnson WE, Parker HS, Jaffe AE, Storey JD (2012). The sva package for removing batch effects and other unwanted variation in high-throughput experiments. Bioinformatics.

[24] Zou H, Hastie T (2005). Regularization and variable selection via the elastic net. J R Stat Soc Ser B Stat Methodol.

[25] Langfelder P, Horvath S (2008). WGCNA: an R package for weighted correlation network analysis. BMC Bioinformatics.

[26] Durinck S, Moreau Y, Kasprzyk A, Davis S, De Moor B, Brazma A (2005). BioMart and Bioconductor: a powerful link between biological databases and microarray data analysis. Bioinforma Oxf Engl.

[27] Durinck S, Spellman PT, Birney E, Huber W (2009). Mapping Identifiers for the integration of genomic datasets with the R/bioconductor package biomaRt. Nat Protoc.

[28] Benita Y, Cao Z, Giallourakis C, Li C, Gardet A, Xavier RJ (2010). Gene enrichment profiles reveal T-cell development, differentiation, and lineage-specific transcription factors including ZBTB25 as a novel NF-AT repressor. Blood.

[29] Jones MJ, Islam SA, Edgar RD, Kobor MS (2015). Adjusting for cell type composition in DNA methylation data using a regression-based approach.

[30] Shannon CP, Hollander Z, Wilson-McManus J, Balshaw R, Ng R, McMaster R, et al. White Blood Cell Differentials Enrich Whole Blood Expression Data in the Context of Acute Cardiac Allograft Rejection. Bioinforma. Biol. Insights. 2012;49. 10.4137/BBI.S9197 PMC3329187 22550401

