# Supplementary figures and images for: Enumerateblood – an R package to estimate the cellular composition of whole blood from Affymetrix Gene ST gene expression profiles
Source: BMC Genomics. 2017 Jan 6;18:43. doi: 10.1186/s12864-016-3460-1 (PMC5219701; doi:10.1186/s12864-016-3460-1)

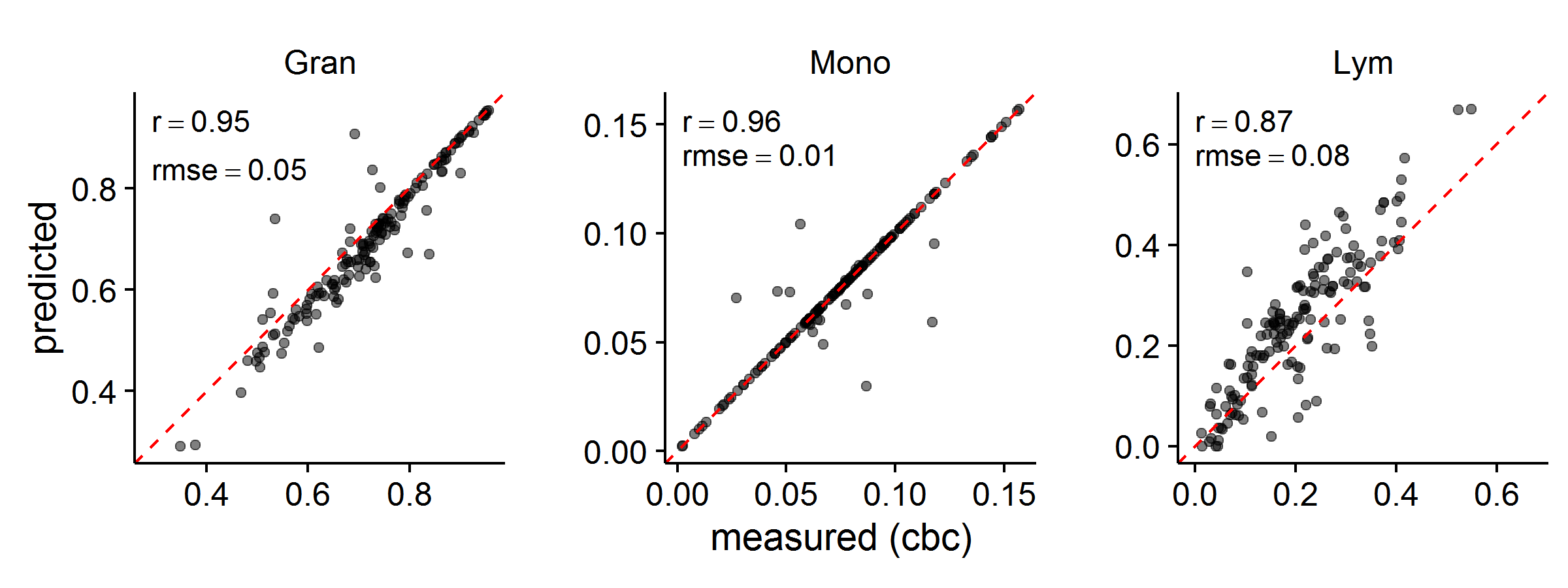

Supplement: Additional file 1: Figure S1. — DNA methylation-derived composition vs. CBC/Diffs. Predicted proportions were obtained by applying the ‘estimateCellCounts’ function from the ‘minif R package to peripheral blood derived DNA methylation profiles in the Rapid Transition Program (RTP) cohort and plotted against cell proportions obtained from CBC/Diffs. The sum of the predicted B, CD4+ T, CD8+ T and NK cell proportions is compared to the total lymphocyte proportions from the CBC/Diffs. For each cell type, Spearman’s rank correlation (ρ) and the root mean squared error (RMSE) are reported. (PNG 47 kb) [file 12864_2016_3460_MOESM1_ESM.png]

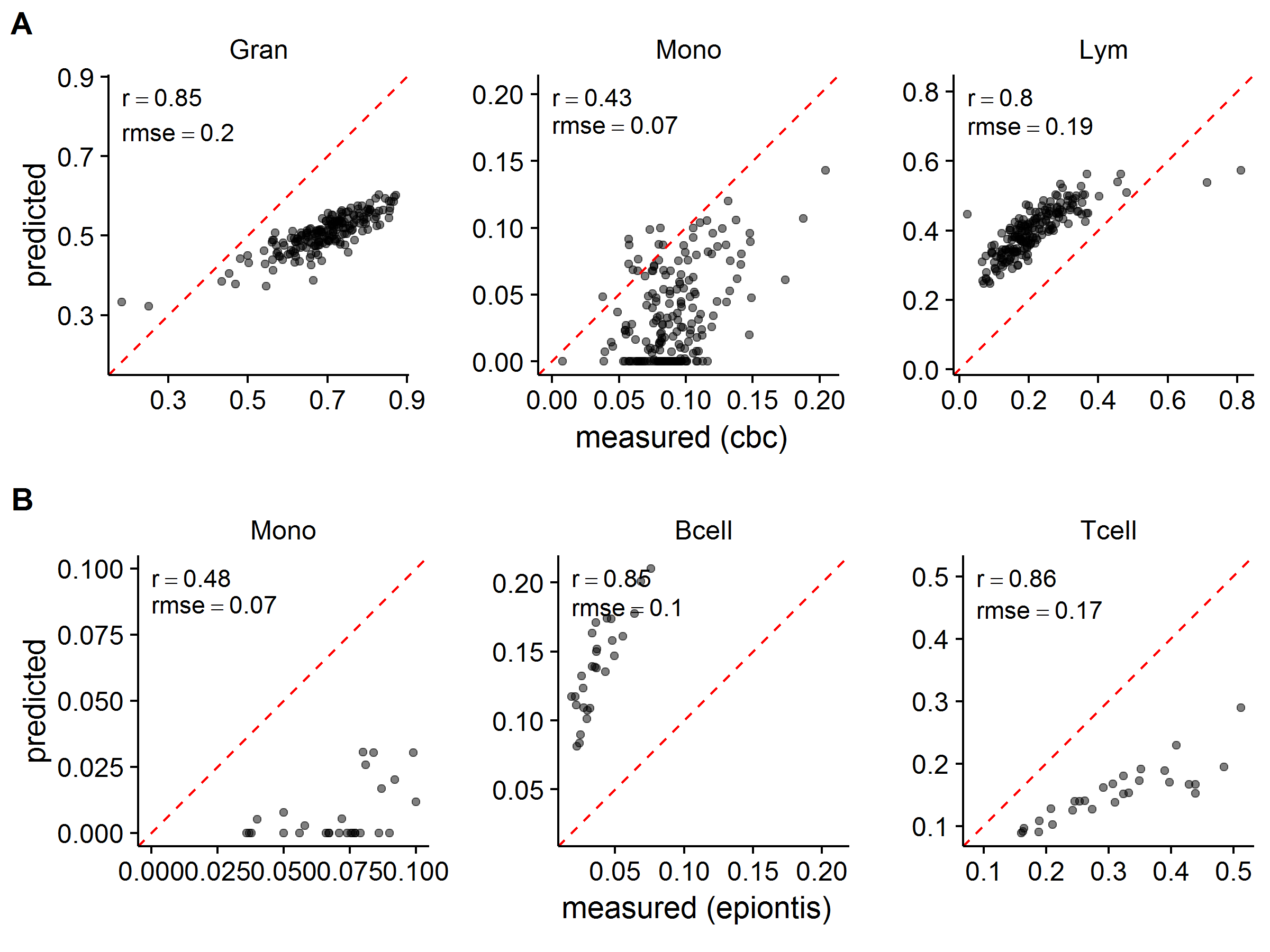

Supplement: Additional file 3: Figure S2. — Performance of Abbas et al. method in our validation datasets (Gene ST). Predicted cell proportions (using the method from Abbas et al.) are plotted against the cell proportions obtained from CBC/diffs (A; CHFP cohort) or a cell-type specific DNA methylation cell-typing assay (B; Epiontis asthma cohort). In A, the sum of the predicted B, CD4+ T, CD8+ T and NK cell proportions is compared to the total lymphocyte proportions from the CBC/diffs. The predicted granulocyte and monocyte proportions are directly compared. In B, the sum of the predicted CD4+ and CD8+ T cell proportions is compared to T cell proportion from the Epiontis assay. The predicted monocyte and B cell proportions are directly compared. For each cell type, Pearson’s product–moment correlation (Pearson’s r) and the root mean squared error (RMSE) are reported. (PNG 83 kb) [file 12864_2016_3460_MOESM3_ESM.png]
